# Genetic alterations affect immune contexture of non-small cell lung cancer: Ukrainian study

**DOI:** 10.3389/fmed.2025.1558016

**Published:** 2025-07-30

**Authors:** Denys Kozakov, Nazarii Kobyliak, Sofiia Livshun, Oleksii Seleznov, Olena Koshyk, Alina Matvieieva, Yaroslav Shparyk, Oleksii Kolesnik, Yuliia Moskalenko, Oleksandr Vynnychenko, Roman Moskalenko, Serhii Kropyvko, Anna Khmel, Bogdana Shkarupii, Oksana Sulaieva

**Affiliations:** ^1^Medical Laboratory CSD, Kyiv, Ukraine; ^2^Institute of Molecular Biology and Genetics NASU, Kyiv, Ukraine; ^3^Department of Endocrinology, Bogomolets National Medical University, Kyiv, Ukraine; ^4^Department of Pathology, Kyiv Medical University, Kyiv, Ukraine; ^5^Communal Nonprofit Enterprise of Lviv Regional District “Lviv Regional Oncology Medical Diagnostic Centre”, Lviv, Ukraine; ^6^Denys Clinics, Kyiv, Ukraine; ^7^Department of Oncology, European International University, Kyiv, Ukraine; ^8^Department of Oncology, Sumy State University, Sumy, Ukraine; ^9^Specialized Mammalogical Center, Kyiv, Ukraine; ^10^Audubon Bioscience, Kyiv, Ukraine; ^11^Ukrainian Association of Research Biobanks, Kyiv, Ukraine; ^12^Ukrainian Association for Precision Medicine, Kyiv, Ukraine

**Keywords:** non-small cell lung cancer, adenocarcinoma, genetic alterations, oncogenes, immune contexture, cancer immunity cycle

## Abstract

**Introduction:**

Although the role of various genetic alterations was highlighted among factors affecting the response to immunotherapy in non-small cell lung cancer (NSCLC), the relations between oncogenic driver variants and changes in the cancer immunity cycle are still unclear.

**Aim:**

The study aimed to discover the links between the molecular and immune context of NSCLC.

**Materials and methods:**

This cohort study included 254 cases of NSCLC (193 Lung Adenocarcinomas) (LUAD; 76%), and 61 squamous cell carcinomas (SCC; 24%), with pathology reports and next-generation sequencing (NGS) data available. First, the rate and spectrum of genetic alterations were assessed in the Ukrainian cohort. Second, we uncovered the relationship between the oncogenic driver mutations and PD-L1 expression in NSCLC. Finally, T-cytotoxic lymphocytes (CD8^+^) and tumor-associated macrophages (CD163^+^) were evaluated in samples with *EGFR* and *KRAS* mutations, *ALK* rearrangements and LUAD with no genetic findings. Immune desert, immune excluded and inflamed types of tumor immune microenvironment (TME) were defined according to the cancer immunity cycle.

**Results:**

More than half (52%) of the observed NSCLC cases harbored single (48.03%) or concomitant (3.94%) genetic alterations in oncogenes. The Ukrainian cohort demonstrated a high rate of *EGFR* (18.5%) and *ALK* rearrangements (9.4%) with a relatively moderate frequency of *KRAS* mutations (16.9%). NSCLC tumors with alterations in *EGFR* and *ALK* demonstrated a high incidence of PD-L1 expression and specific immune contexture. The number of CD8^+^ cells varied significantly between oncogene-driven and wild-type LUAD (*p* = 0.019). Non-oncogene-addicted NSCLC demonstrated the prevalence of Inflamed TME rich in CD163^+^ macrophages. In contrast, over half of *EGFR* mutant LUAD cases possessed immune desert TME type, while *ALK*-rearranged and *KRAS* mutant NSCLC showed mostly immune excluded TME.

**Conclusion:**

The high rate of PD-L1 expression in NSCLC driven by *EGFR* and *ALK* alterations was accompanied by a prevalence of low immunogenicity with a shift toward ID TME in *EGFR* mutant tumors and IE TME in *ALK-*rearranged and *KRAS* mutant NSCLC. Further discovery of mechanisms affecting tumor immune contexture is needed for tailoring patient management in line with particular mechanisms of immune evasion.

## Introduction

Non-small cell lung cancer (NSCLC) remains the leading cause of death among various malignancies globally (1). The exploration of the molecular landscape of NSCLC has revealed numerous clinically actionable genetic alterations defining a wide spectrum of biomarker-directed targeted therapies, tailoring personalized patient management and improving outcomes (2). An alternative approach relying on reactivating patient immune response against cancer cells facilitated the implementation of immunotherapy that has revolutionized NSCLC treatment (3). The discovery of CTLA-4 and PD-1/PD-L1 at the beginning of the 21^st^ century resulted in practice-changing implementation of immune checkpoint inhibitors (ICI) (4). Several studies demonstrated the efficiency of immunotherapy in patients with NSCLC (5, 6), emphasizing the clear benefits of ICI for NSCLC patient survival and quality of life. Immunotherapy has become a part of standard treatment for patients with NSCLC (7). The benefits of ICI have now been recognized in the early stages of the disease, resulting in the durable reinvigoration of the patient’s immune system and the emergence of long-term responders (4, 8). Nevertheless, not all patients respond to immunotherapy, and only a few patients achieve long-term survival under ICI application.

The role of various genetic alterations was highlighted among factors that affect response to immunotherapy (9). Existing evidence suggests a severe limitation to the efficacy of ICI in patients with *EGFR*-mutant NSCLC, especially after developing resistance to tyrosine kinase inhibitor (TKI) (10, 11). Similarly, real-world data demonstrated weak response to ICI in *ALK*-positive NSCLC patients, particularly compared to those treated with approved *ALK* TKIs (12). Although various mutations are associated with upregulation of PD-L1 expression in tumor cells, the mechanisms of decreased efficacy of ICI on *EGFR-and ALK*-driven NSCLC remains unclear (13, 14). Alternatively, *KRAS* mutations in NSCLC were shown to coincide with a better response to ICI, which might be related to T-cell infiltration or expression of PD-L1 (15). Besides, most *KRAS*-mutant tumors are found in tobacco smokers with corresponding signatures and a high tumor mutational burden (TMB) (16). Hereby, the interplay between tumor molecular profile and response to ICI is still under debate, being actively researched and varying significantly between different populations (17).

Genetic alterations in oncogenic drivers such as *EGFR*, *ALK*, *KRAS*, *BRAF*, *MET* and others can modify immunogenicity and impact the tumor microenvironment (TME) (18). Recent studies highlighted the importance of CD8^+^ tumor-infiltrating lymphocytes and other immune cells in defining the response to ICI (19, 20). Indeed, CD8^+^ cells are a crucial component of antitumor immune response and a backbone of currently approved cancer immunotherapies (21). An entire framework of sequential events generating anti-tumor immune response is embedded in the concept of the cancer-immunity cycle, which defines specific mechanisms of tumor-immune coevolution and its immunophenotypical repercussions. Furthermore, a high infiltration by CD8^+^ cells is prognostically beneficial in patients with NSCLC and many other cancers (22, 23). However, little is known about the impact of targetable oncogenic driver mutations on the cancer-immunity cycle in NSCLC. This defines an interest in associating oncogenic driver variants with tumor immune contexture, which can help to identify mechanisms of tumor immune evasion and predict sensitivity to various classes of immunotherapy.

The study aimed to assess the rate and spectrum of genetic alterations in NSCLC in the Ukrainian cohort and to discover the links between molecular and immune contexture of NSCLC.

## Materials and methods

### Setting and participants

A total of 254 patients were enrolled in this cohort study. Descriptive information on the patients is presented in Table 1. The observed cohort consisted of 157 males (61.8%) aged 61 (54.8–66.3), and 97 females (38.2%) aged 56 (46–67). 193 patients had Lung Adenocarcinoma (LUAD; 76%), and 61 patients were diagnosed with Squamous cell carcinoma (SCC; 24%).

**Table 1:** Characteristics of patients of the study.

Parameters	Values	*p*
Number of participants	254	
Sex		
Men	157 (61.8%)	
Women	97 (38.2%)	
Diagnosis		
LUAD	193 (81.6%)	
SCC	61 (17.3%)	
Age, years		0.001
Men	61 (54.8–66.3)	
Women	56 (46.0–67.0)	
Age at diagnosis, years		0.008
LUAD	60 (49.0–66.0)	
SCC	62 (55.7–69)	

Data presented as Me (QI–QIII) or *n* (%).

The study included three phases addressing three particular research questions. In the first stage, we assessed the rate and spectrum of genetic alterations in known oncogenes in NSCLC in the Ukrainian population. This stage included 254 NSCLC patient tumors with pathology reports and next-generation sequencing (NGS) data available. All the enrolled cases underwent NGS testing with a 10-gene panel for detecting single nucleotide variants (SNVs) and indel in *EGFR, ALK, ROS1, RET, KRAS, NRAS, PIK3CA, BRAF, ERBB2* and *MET*; fusions involving *ALK, ROS1* and *RET*, as well as exon 14 CNV analysis in *MET* gene (Amoy Dx Essential panel, China) using NextSeq 550Dx (Illumina, US).

In the second stage, we assessed the relationship between the oncogenic driver mutations and PD-L1 expression in NSCLC. This stage included 180 formalin-fixed paraffin-embedded (FFPE) tumor samples of patients who had NGS testing performed and PD-L1 immunohistochemical (IHC) results available.

In the third stage, 40 samples from the primary cohort were analyzed *via* IHC to assess the immune contexture of LUAD harboring targetable genetic alterations in oncogenes (10 samples with *EGFR* mutations, 10 samples with *KRAS* mutations, 10 samples with *ALK* rearrangements) compared to LUAD tumors with no genetic findings (WT).

### Molecular analysis

Extraction of DNA was performed using Omega Bio-tek E.Z.N.A.® FFPE DNA Kit (US). The quantity of DNA was assessed via DeNovix dsDNA Broad Range Assay (US). DNA fragmentation levels were measured with ArcherDX PreSeq DNA QC Assay (US). AmoyDx Essential NGS Panel from AmoyDx (China) was used to test FFPE samples *via* NGS. Library preparation was performed according to the manufacturer’s protocol. No less than 30 ng of DNA input material was used for library preparation. Normalization to 4 nM concentration and sample pooling was performed after quantifying each library using Roche KAPA Library Quantification Kit (Switzerland) and fragment size analysis via Agilent TapeStation (US).

Sequencing was performed using the Illumina NextSeq 550 Mid-Output Kit on an Illumina NextSeq 550Dx (US) platform. NGS data analysis and annotation of genetic variants (SNV, indel, gene fusions) was performed using AmoyDx ANDAS server analysis module ADXLC10, version 3.3.0 (AmoyDx).

### Methodology of immune contexture assessment in LUAD

In order to uncover the possible mechanisms of immune evasion in LUAD, T-cytotoxic lymphocytes (CD8^+^) and tumor-associated macrophages (CD163^+^) were visualized via IHC in samples with *EGFR* and *KRAS* mutations, *ALK* rearrangements and samples with no genetic findings (10 samples per group).

Several types of TME were defined: Immune desert (ID), immune excluded (IE) and inflamed (Inf). This was done to assess tumor-host interplay within the cancer immune cycle (24–26). The ID type reflects a lack of pre-existing immunity and demonstrates a low number of T-cells inside and around the tumor. The IE type shows prominent peritumoral infiltration but few intratumoral T-lymphocytes and is indicative of a failure in T-cell trafficking. The Inf TME type displays severe lymphocytic infiltration, reflecting the activation of antitumor T-cells with impaired antitumor activity (27). The number and spatial distribution of CD8^+^ cells were analyzed with respect to heterogeneity within tumor clusters (TC) and tumor stroma (TS) in 10 different fields of view (28). Next, the number of CD163^+^ cells corresponding to M2 macrophages was assessed at TC and TS in samples with respect to every TME type. The count of immune cells was recalculated per 1 mm^2^.

### Immunohistochemistry

For immunohistochemistry, 4 μm thick sections were cut, mounted on positively charged slides, and stained according to the manufacturer’s protocol. Visualization of anti-tumor T-cytotoxic lymphocytes was done using antibodies to CD8 (Clone C8/144B, Agilent, United States). Assessment of M2-macrophages was done with CD163 antibodies (Cell Marque, Clone MRQ-26). Additionally, PD-L1 (Dako, 22C3) expression was assessed in all of the selected cases to investigate tumor immune escape mechanisms. PD-L1 expression was assessed *via* Tumor Proportion Score (TPS). PD-L1 positivity was considered in the at TPS ≥ 1%. Tumors with PD-L1 expression levels of 1–49% were annotated PD-L1-low positive, and PD-L1 expression of 50% and higher were considered PD-L1 high. Two independent pathologists conducted histological and IHC evaluations for each case.

### Statistical analysis

Statistical analysis was conducted using MedCalc® Statistical Software version 22.016^1^ (MedCalc Software Ltd., Ostend, Belgium; 2023) and GraphPad Prism [GraphPad Prism Version 10.4.0^2^ (621 GraphPad Software, San Diego, California, United States)]. Descriptive statistics were provided as Median and interquartile range (IQR; Q_I_ – Q_III_). The Kruskal-Wallis test was applied to compare continuous variables between subgroups. Categorical data were assessed as frequency (%). χ^2^ or Fisher’s exact tests were used to compare frequencies. The *p* value of < 0.05 was considered statistically significant.

## Results

Aligned with the world NSCLC statistics, there were significant gender differences between histological subtypes of NSCLC (*p* = 0.002). LUAD cohort included 86 females (44.6%) and 107 males (55.4%). Female patients were diagnosed with cancer at a younger age [55 (46–67) years old] compared to males [60 (55.0–62.8) years old], although these differences were not significant (*p* = 0.09). In contrast, SCC was more prevalent in males (50; 82.0%) than females (11; 18.0%).

### Rate of genetic alterations in Ukrainian patients with NSCLC

**Figure 1:**
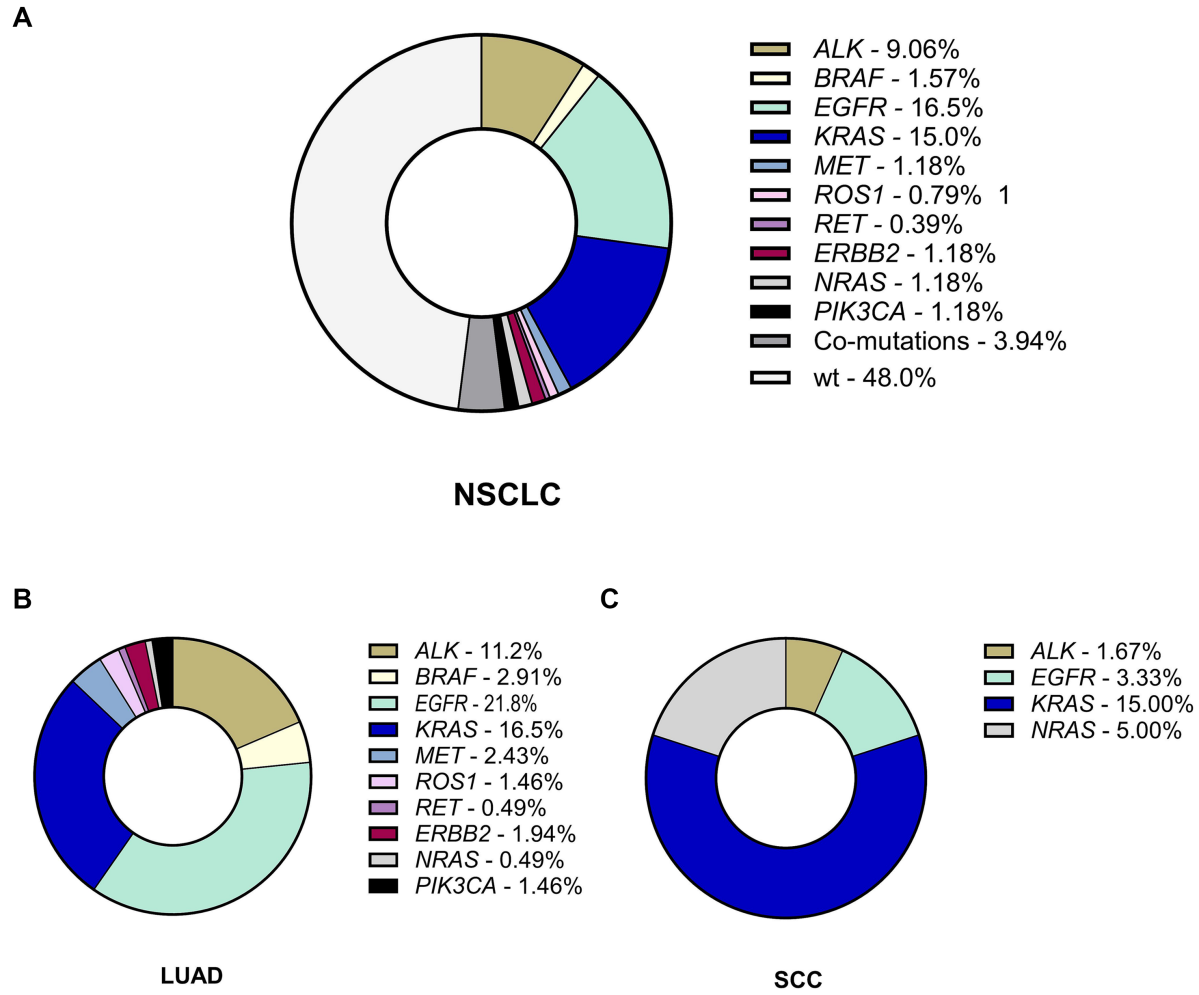
The incidence and spectrum of genetic alterations in NSCLC. **(A)** The frequency of genetic alterations in the whole Ukrainian cohort of patients with NSCLC. **(B)** The spectrum and rate of genetic findings in LUAD. **(C)** The spectrum and rate of genetic findings in SCC. LUAD, lung adenocarcinoma; SCC, squamous cell carcinoma.

More than half of the observed NSCLC cases harbored genetic alterations (132 out of 254 patients; 52%) in oncogenes. Among the observed cohort, 122 patients harbored a single alteration in oncogene (48.03% of the cohort), and 10 patient samples had co-existing mutations in one or more genes comprising 7.58% of oncogene-driven NSCLC cases and 3.94% of the whole sample size (Figure 1). All the co-occurring mutations were found in LUAd cases.

Aligning with previous reports, the rate of *EGFR* genetic variants was much higher in females (31 of 97 patients; 32.0%, Supplementary Table S1) compared to males (16 of 157 patients; 10.2%; *p* < 0.001). Similar sex differences were typical for *ALK* rearrangements (*p* = 0.014). *ALK* rearrangements were associated with a younger age of onset (48; 44–53) compared to WT-NSCLC (61; 53.0–67.5). Although the rate of *KRAS* mutations was almost as twice as high in males with NSCLC compared to females (32 of 157 cases, 20.4% in males *vs* 11 of 97 cases; 11.3% in females), this difference was not found significant (*p* = 0.084). Other genetic alterations also did not demonstrate statistically significant differences (Supplementary Table S2).

### Rate of genetic alterations in LUAD and SCC

The rate of genetic alterations in LUAD (116 cases with alterations of 193 LUADs; 60.1%) was almost three times higher than that of SCC (16 of 61; 26.2% in SCC; *p* < 0.001), demonstrating higher frequency of *EGFR* and *ALK* genetic findings (Supplementary Table S2). *EGFR* mutations were found in 47 patients (18.5%), 45 of which had LUAD (23.3%) and 2 SCC (3.3%; p < 0,001). Similarly, *ALK* mutations found in 24 patients (9.4%) were also more common in LUAD (23 of 194 cases in LUAD; 11.9%) than SCC (1 of 61 cases; 1.6%; *p* = 0.012). The most common fusion partner for *ALK* was *EML4*, although we found 2 NSCLC cases with *DCTN1* (exon 26)-*ALK* (exon 20) fusions.

Alterations in *KRAS*-oncogene were detected in 43 patients (16.9%), who represented single gene mutations (*n* = 38) and co-occurring mutations in *KRAS* and other genes (*n* = 5). The rate of *KRAS* mutations was comparable among NSCLC of different histology reaching 17.6% in LUAD (34 of 193) and 14.8% in SCC (9 of 61; *p* = 0.689). Finally, 6 patients with LUAD (3.1%) harbored *BRAF* mutations, 5 (1.97%)—*MET* alterations, 3 (1.18%)—*ROS1* rearrangements, 4 (1.57%)—*ERBB2* mutations, 1 (0.39%)—a *RET* gene fusion. The frequency of alterations in *BRAF, MET, ROS1, ERBB2*, *PIK3CA* and *RET* genes was not linked to gender or histological type of NSCLC (Supplementary Tables S1, S2). Besides 4 patients (1.57%) harbored *NRAS* mutation as a single gene alteration (*n* = 1) or as a co-existing mutation (*n* = 3), and these alterations were more common in SCC (*p* = 0.044). Thus, the Ukrainian cohort demonstrated a relatively high rate of *EGFR* and *ALK* alterations in LUAD with a moderate frequency of *KRAS* mutations compared to other European cohorts.

### Spectrum of oncogene-driven genetic alterations in NSCLC

Analyzing the spectrum of genetic variants in *EGFR* gene, we found 50 primary and secondary *EGFR* mutations in 47 NSCLC patients (including 3 mutations co-occurring with other *EGFR* alterations) (Table 2). The most prominent hotspots for *EGFR* mutation were exons 21 and 19 including p.Leu858Arg (44.0%) and exon 19 deletions (38.0%), representing the main anti-*EGFR* TKI susceptibility mutations. Rare mutations in exon 18 accounted for 14% of *EGFR* genetic findings. Among them, a rare mutation p.Leu718Gln associated with resistance to osimertinib (29) was found in one patient (2%). An exon 20 insertion was detected in one patient and represents 2% of *EGFR* mutation spectrum in this cohort. A secondary p.Thr790Met variant associated with acquired resistance to first-and second-generation anti-*EGFR* TKI was also detected in one patient (2% of *EGFR* mutation spectrum). Among the observed *EGFR* alterations 2 mutations found in SCC included L858R and deletion in exon 19, while LUAD possessed a much wider spectrum of *EGFR* mutations.

**Table 2:** The spectrum of *EGFR* mutations in NSCLC.

Type of *EGFR* mutation	Exon	Number of cases	Rate among *EGFR* variants	Rate in cohort
c.709_710delinsTA (p.A237Y)	18	4	8.0%	1.57%
c.2125G>A (p.Glu709Lys)	18	1	2.0%	0.39%
c.2156G>C* (p.Gly719Ala)	18	1	2.0%	0.39%
c.2153T>A* (p.Leu718Gln)	18	1	2.0%	0.39%
c.2235_2249del (p.Glu746_Ala750del)	19	11	22.0%	4.33%
c.2236_2250del (p.Glu746_Ala750del)	19	5	10.0%	1.97%
c.2237_2255delinsT (p.Glu746_Ser752delinsVal)	19	1	2.0%	0.39%
c.2240_2257del (p.Leu747_Pro753delinsSer)	19	2	4.0%	0.79%
c.2311_2319dup (p.Asn771_His773dup)	20	1	2.0%	0.39%
c.2369C>T* (p.Thr790Met)	20	1	2.0%	0.39%
c.2573T>G (p.Leu858Arg)	21	22	44.0%	8.66%

^*^Corresponds to 3 co-existing mutations found in combinations with other mutations in EGFR gene.

There were 5 cases with co-occurring primary and secondary mutations in *EGFR* or other genes. One of them combined *EGFR* L858R with two mutations of resistance (T790M and L718Q), and another patient demonstrated a complex of p.Glu709Lys combined with p.Leu747_Pro753delinsSer. There was a case of co-existing p.Glu709Lys and p.Gly719Ala alterations in *EGFR*. Besides we found one case with co-mutations in *EGFR* and *BRAF* genes (*EGFR* L858R + *BRAF*c1799T>A) and one NSCLC harboring *EGFR* c.2235_2249del (p.Glu746_Ala750del) combined with *MET* amplification.

The spectrum of *KRAS* point mutations comprised single nucleotide variants and indels in clinically relevant exons 2 and 4. p.G12C substitution, associated with sensitivity to *KRAS* small molecule inhibitors, was the most common and comprised more than one-fourth (12 of 43; 27.91%) of mutations in *KRAS* (Table 3). Among exon 4 mutations, codon 146 substitution was detected in one patient (2.56%) and a point p.Lys117Arg mutation was detected in 2 patients with NSCLC (5.12%).

**Table 3:** The spectrum of *KRAS* mutations in Ukrainian patients with NSCLC.

Type of *KRAS* mutation	Number of cases	Rate among *KRAS* variants	Rate in cohort
c.35G>C (p.Gly12Ala)	3	6.98%	1.18%
c.34G>A G12S	1	2.33%	0.39%
c.34_35delinsTT (p.Gly12Phe)	1	2.33%	0.39%
c.34G>T (p.Gly12Cys)	12	27.91%	4.72%
c.35G>A (p.Gly12Asp)	10	23.26%	3.94%
c.35G>T (p.Gly12Val)	10	23.26%	3.94%
c.37G>T (p.Gly13Cys)	2	4.65%	0.79%
c.38G>A (p.Gly13Asp)	2	4.65%	0.79%
c.35A>G (p.Lys117Arg)	1	2.33%	0.39%
c.183A>C (p.Q61H)	1	2.33%	0.39%

Interestingly, 5 cases with *KRAS* mutations demonstrated co-existing genetic alterations in other genes. c.350A>G (p.Lys117Arg) in *KRAS* co-occurred with c.1781A>G (p.Asp594Gly) in *BRAF* and EML4 (exon13) → *ALK* (exon20) translocation in metastatic LUAD. Besides we found 3 cases with co-occurring mutations in *KRAS* (G12S) and *NRAS* (Q61R), one case harboring co-existing mutations in *KRAS* (c.35G>A, p.Gly12Asp) and *ERBB2* (c.2440C>T; p.Arg814Cys), and one case demonstrating co-occurrence of *KRAS* mutation (G12C) with MET substitution (c.2975C>T; p.Thr992Ile). In addition, one sample combined *KRAS* mutation (c.35G>C; p.Gly12Ala) with *ROS1*-rearrangement [*ROS1* (exon33)–SYNPO2 (exon2)].

### PD-L1 expression in oncogene-addicted NSCLC

**Figure 2:**
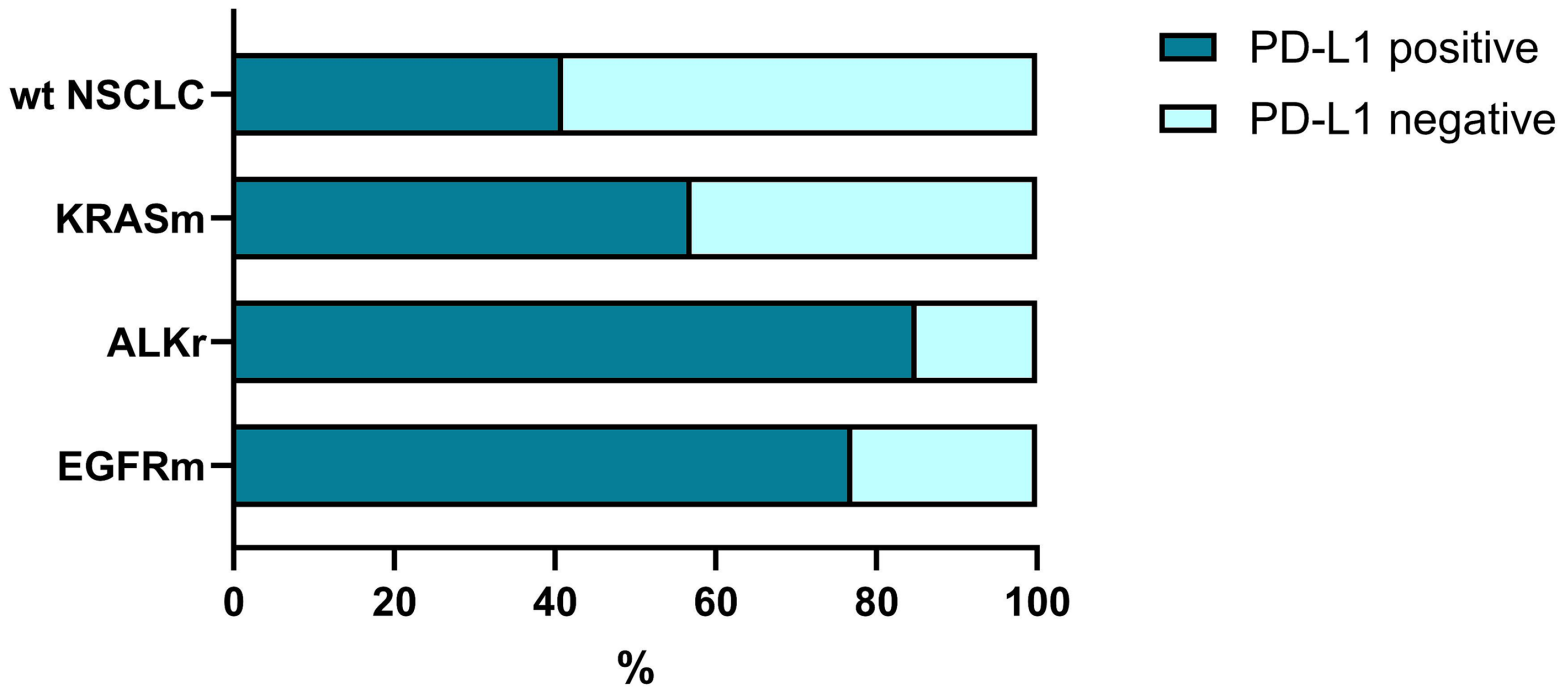
The rate of PD-L1 expression in oncogene-addicted and non-oncogene-addicted NSCLC. Tumors with genetic alterations in *EGFR* and *ALK* demonstrated were prone to PD-L1 expression that reached 85–87% of all carcinomas. In contrast, non-oncogene addicted NSCLC demonstrated a significantly lower rate of PD-L1 expression.

Oncogene-addicted NSCLC demonstrated a significantly higher rate of PD-L1 expression (*p* < 0.001, Figure 2). Among 180 cases with known genetic alterations and PD-L1 expression, 96 were PD-L1 positive (53.3%) and 84 cases (46.7%) did not demonstrate PD-L1 expression. PD-L1 expression with TPS 1–49% was found in 34.4% (62 of 180) and high PD-L1 expression was identified in 18.9% (34 of 180) NSCLC. We found no significant differences in PD-L1 expression between LUAD and SCC (*p* = 0.076; Supplementary Table S3).

Among this set 91 cases were oncogene addicted, and about 2/3 of them (60; 65.9%) demonstrated PD-L1 positivity (Figure 2). In contrast, among non-oncogene addicted NSCLC, only 40.4% (36 of 89) showed positive expression of PD-L1 while the rest of the cases were PD-L1 negative (53 of 89 wild-type cases, 59.6%).

At the same time, tumors harboring *EGFR* mutations demonstrated a higher rate of PD-L1 positivity compared to NSCLC with no genetic findings (*p* = 0.029), specifically in patients with LUAD (*p* = 0.015), but not SCC (*p* = 0.441; Supplementary Table S4).

Similarly, *ALK*-positive NSCLC was associated with a higher expression of PD-L1 (p = 0,048) in both PD-L1 low and PD-L1 high groups (Supplementary Table S3). At the same time, there was no link between *KRAS* mutation status and PD-L1 expression within the cohort (Figure 2; Supplementary Table S4).

Thus, NSCLC tumors with genetic alterations in *EGFR* and *ALK* demonstrated a high prevalence of PD-L1 expression reflecting a predisposition to oncogene-driven immune escape. The concept of the cancer-immunity cycle was applied for further analysis of the tumor immune landscape with regard to genetic changes.

### Links between genetic alterations and cancer immunity cycle in lung adenocarcinoma

**Figure 3:**
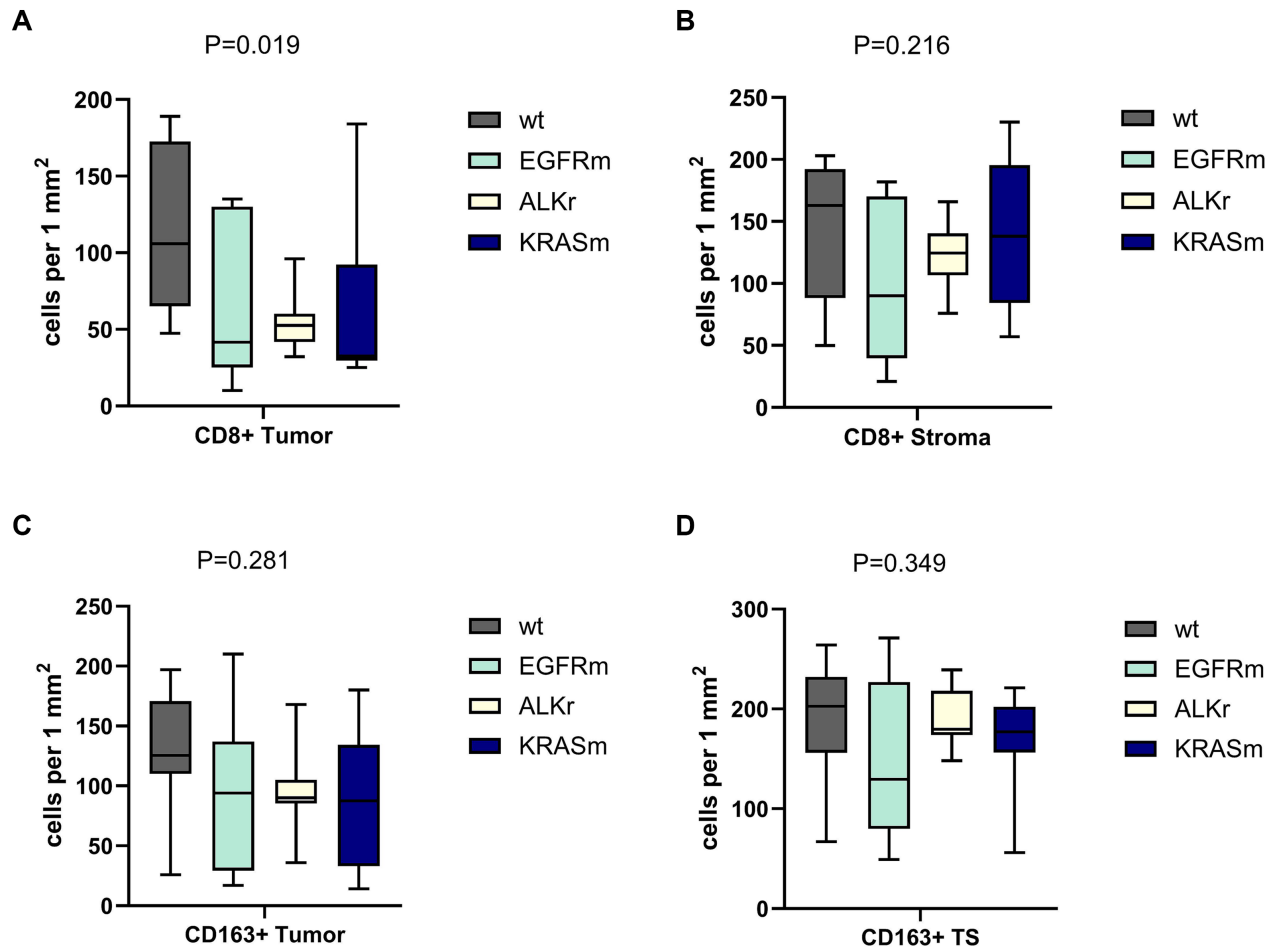
Density of CD8^+^ and CD163^+^ immune cell presence within tumor and tumor stroma of wild-type (WT), *EGFR* mutant, *ALK*-rearranged, and *KRAS* mutant LUAD. Panels **(A,B)** demonstrate the number of CD8^+^ cells within the tumor and in the peritumor stroma, respectively. Panels **(C,D)** showed the amount of CD163^+^ macrophages within the tumor and in the peritumor stroma, respectively. Oncogene-addicted LUAD demonstrated a significantly lower number of CD8^+^ cells infiltrating the tumor.

In this study, oncogene-driven LUAD demonstrated distinguishable peculiarities of tumor-host immunity interplay. The density and distribution of tumor-infiltrating lymphocytes (TILs) differed depending on tumor genetic findings. The number of intra-tumorous CD8^+^ cells varied significantly between oncogene-driven and wild-type LUAD (*p* = 0.019). Wild-type LUAD demonstrated a higher number of tumor-infiltrating CD8^+^ T-cells, while in tumors with *EGFR* and *ALK* alterations, the number of CD8^+^ T-cells was much lower (Figure 3), and *KRASm* LUAD was heterogeneous in terms of tumor-infiltrating CD8^+^ cells count. This finding correlated with the prevalence of ID tumor microenvironment in *EGFR* mutant LUAD and IE immunophenotype among *ALK*-rearranged and *KRAS* mutant adenocarcinomas.

**Figure 4:**
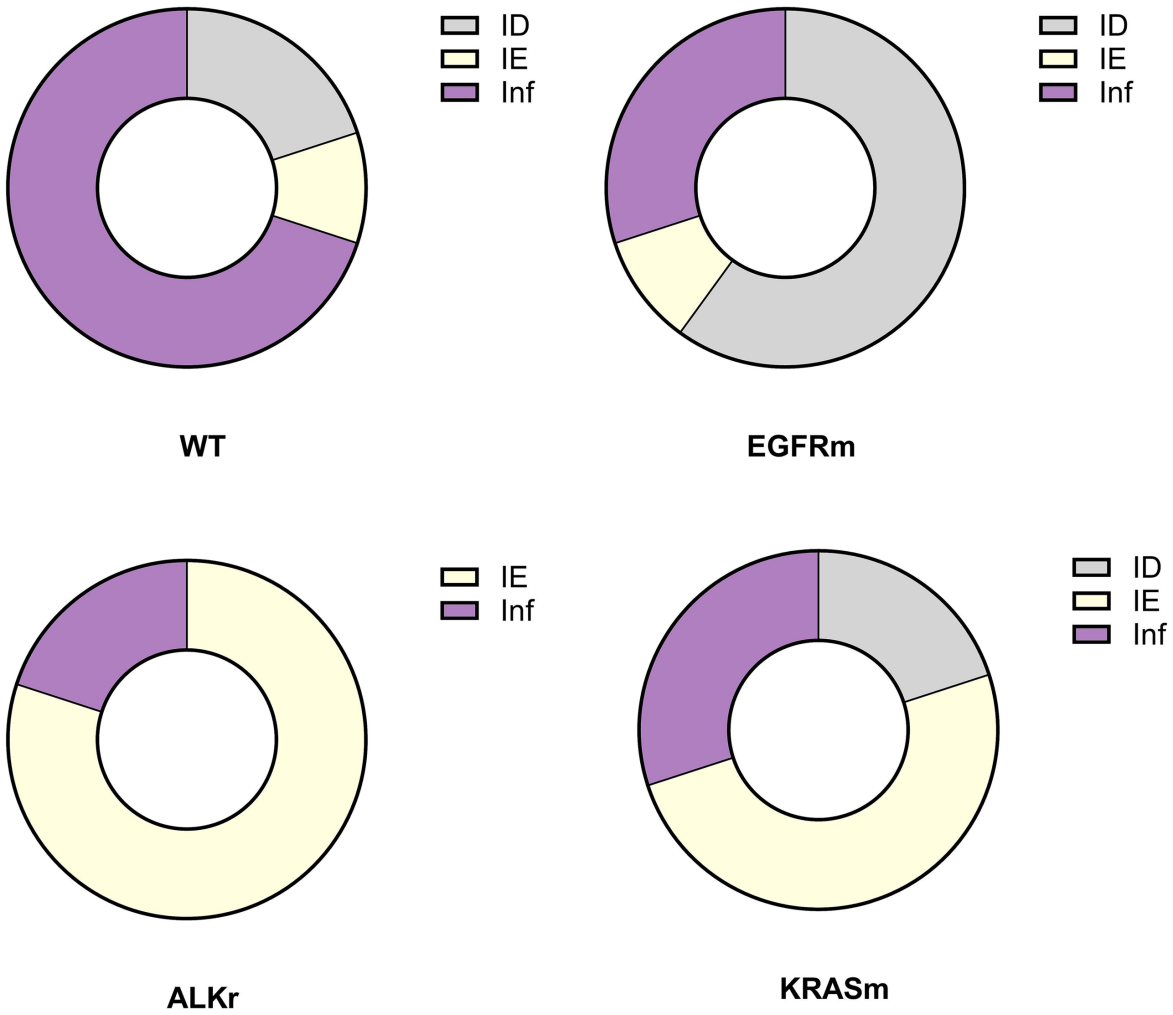
Prevalence of immune phenotypes in tumors with no detected genetic variants (wild-type, WT), *EGFR* mutant (*EGFR*m), *ALK*-rearranged (*ALK*r), and *KRAS* mutant LUAD (*KRAS*m).

Indeed, genetic alterations in oncogenes were linked to the prevalence of various TME types in LUAD (*p* = 0.002). Non-oncogene-addicted NSCLC demonstrated the prevalence of Inflamed TME (Figure 4). In contrast, over half of *EGFR* mutant LUAD cases demonstrated immune desert TME type, 30% possessed immune inflamed TME and 10% had an immune excluded type. In contrast, adenocarcinoma with *ALK* rearrangement demonstrated mostly IE-TME, with few cases having Inflamed TME. *KRASm* NSCLC were the most heterogenous demonstrating 50% cases with IE phenotype and the rest were of ID and Inflamed TME.

**Figure 5:**
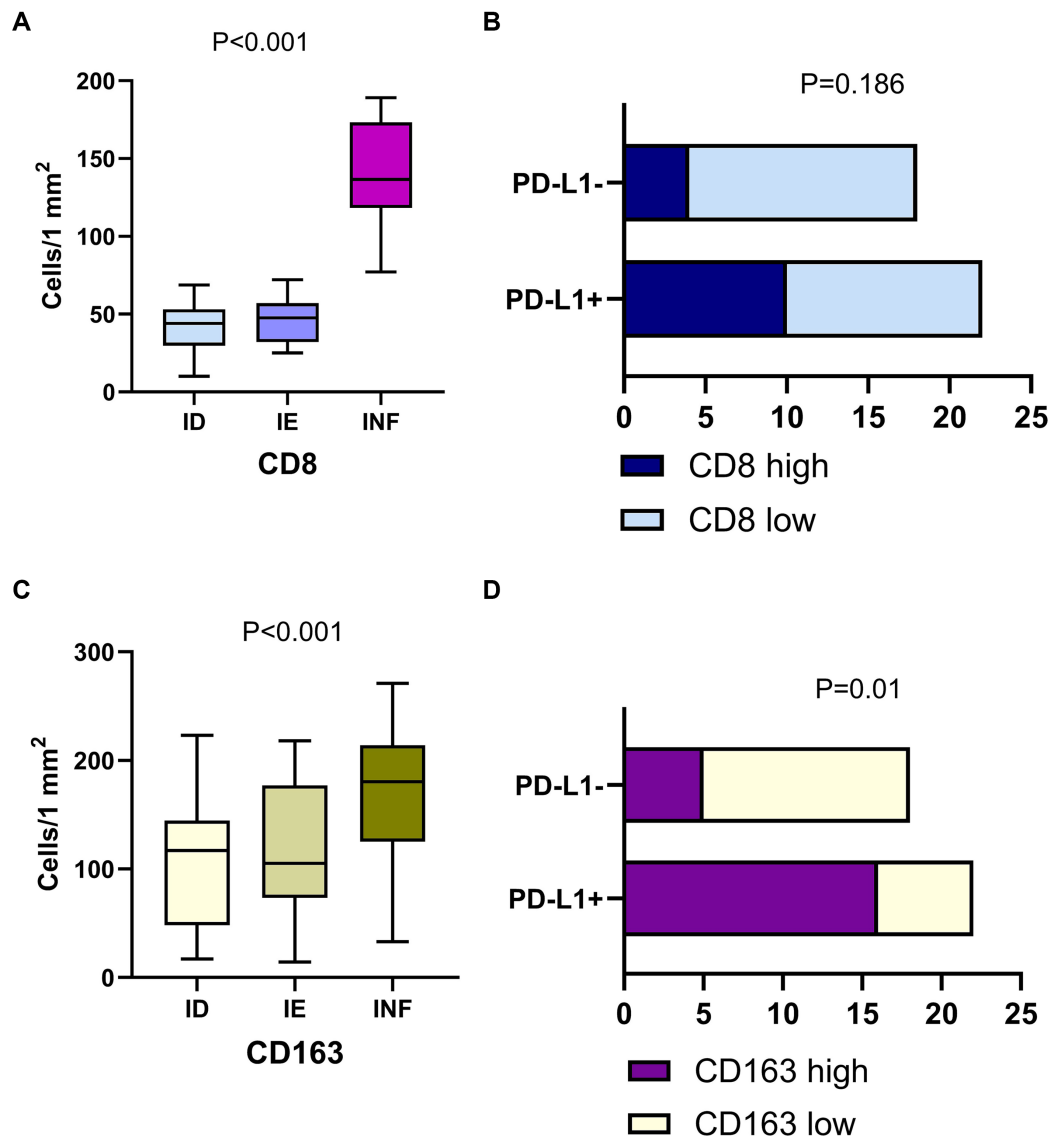
Immune cells in LUAD of with different immune phenotype and their interplay with PD-L1 expression. Panels **(A,C)** demonstrate the count of CD8^+^ cells and CD163 macrophages in LUAD of the immune desert (ID), immune excluded (IE) and Inflamed (INF) type of tumor microenvironment. Panels **(B,D)** show the link between PD-L1 expression and density of CD8^+^ cells and CD163^+^ macrophages. Positive tumor PD-L1 status is related to a high density of CD163^+^ cells, but not CD8^+^ cells.

Notably, the tumor-associated macrophage (TAM) number was significantly higher than CD8^+^ cells in LUAD. The count of TAMs was comparable in ID and IE cases but was significantly increased in tumors with immune-inflamed TME (*p* < 0.001; Figure 5). The high number of CD163^+^ cells within the tumor was associated with positive PD-L1 status (*p* = 0.01), although there was no statistically significant link between the number of CD8^+^ cells and PD-L1 expression.

Thus, here we report the prevalence of ID TME in *EGFR* mutant tumors and IE TME in *ALK-*rearranged and *KRAS* mutant LUAD, while wild-type LUAD mostly displayed immune-inflamed TME type. Inflamed TME was associated with higher M2 macrophages affecting the rate of PD-L1 expression. This data suggests an interaction between genetic alterations in LUAD and molecular mechanisms regulating the cancer-immunity cycle. With these findings, further investigation into the specific mechanisms regulating TME is necessary to apply the observed markers of immune evasion to patient management and diagnostics.

## Discussion

This study first described the molecular landscape of NSCLC in the Ukrainian population. We found a high rate of genetic alterations in LUAD and a significantly lower rate of actionable mutations in SCC. In general, this data aligns with the global reports on the molecular landscape of NSCLC (30, 31).

At the same time, the Ukrainian cohort observed in this study demonstrated peculiar features. First, the rate of alterations in *EGFR* and *ALK* was relatively high, while the incidence of *KRAS* mutations was lower than the average reported frequency worldwide. Various populations demonstrate differences in the spectrum of oncogenic driver mutations. For instance, in an Irish study on 2052 NSCLC patients, actionable genetic alterations were found in 53% of cases, with the highest rate of *KRAS* mutation reported to be 32%, while the rate of *EGFR* mutation was only 8.8%, and *ALK* rearrangements were found in only 2.1% cases (32). Alternatively, in the Indian cohort of 325 patients with NSCLC, the rate of *EGFR* mutation reached 45.8%, and *ALK* rearrangements were found in 11.4% of cases, however, *KRAS* mutations were present in only 10.2% (33). The availability of such population data in cancer research is essential to understanding the ethnic peculiarities of NSCLC, planning clinical trials and introducing novel precision medicines.

The rate of genetic mutation in NSCLC also depends on various extrinsic (tobacco smoking, air pollution, exposure to radiation or occupational) factors as well as intrinsic factors, including population distribution, mean / median age, gender, histological tumor type and stage, etc (34). There are well-known facts on the association of NSCLC genotypes with such factors, e.g., *EGFR* mutations and *ALK* fusions prevail in female patients with adenocarcinoma of younger age and no history of smoking, while *KRAS* mutations are more common in smokers (35). Besides, the role of alterations in genes affecting chromatin remodeling and intercellular crosstalk can facilitate immune evasion mechanisms affecting susceptibility to immunotherapy (9). Importantly, NGS testing affordability is also a factor impacting the rate of detected genetic alterations, especially in developing countries where these expensive services are paid mostly out of pocket (36).

According to this study’s results, alterations in *EGFR* and *ALK* were associated with high PD-L1 expression in NSCLC. The high rate of PD-L1 positive NSCLC aligns with previous data, demonstrating positive PD-L1 status in up to 65% of NSCLC tumors (37). Indeed, the increased rate of PD-L1 positive tumors was previously shown for *EGFR* mutant LUAD (38, 39). Tumors harboring *EGFR* variants can evade immune response by exposing PD-L1 on tumor cells and inhibiting T-cell antitumor activity (40). *EGFR* activation in cancer cells can upregulate PD-L1 through the MAPK signaling pathway that leads to increased activity of ERK1/2 and AKT which facilitate cell proliferation and invasion. However, high expression of PD-L1 in *EGFR* mutant NSCLC, in theory, should make the tumor susceptible to ICI, shaping the possibility to reactivate the immune system against tumor cells. However, as it was previously shown, anti-PD-1/PD-L1 therapy demonstrates limited success in NSCLC with activating *EGFR* and *ALK* genetic alterations (41). Importantly, we found that increased PD-L1 expression was linked to high numbers of M2 macrophages, which is consistent with the data from other cancer types (25, 26).

At the same time, in our study, *KRAS* mutant carcinoma was also found to be heterogeneous with the prevalence of IE TME and less frequent inflamed immune contexture. This data at least partly contradicts the widely accepted concept of high immunogenicity of *KRAS* mutant NSCLC. A recent study illuminated that *KRAS* mutation status in NSCLC positively correlated with TMB, higher neoantigens generation, PD-L1 expression, and T-cell infiltration (16). On the other hand, the impact of *KRAS* mutations on the immunity responses remains disputable in NSCLC patients (42). Moreover, it was shown that *KRAS* status did not affect patients outcomes under nivolumab application (43). Interpreting the prevalence of various TME types in oncogene-driven NSCLC, it seems essential to understand the bidirectional nature of the interplay between the mutational landscape and immune contexture of tumors. On one hand, genetic alterations can be associated with the expression of neoantigens improving the recognition of tumor cells and activation of the cancer immunity cycle. On the other hand, tumor-secreted exosomes, hypoxia, and acidic milieu can promote immunosuppressive TME (42). The complexity of tumor-host interplay also relies on chronological dimension, cancer cells evolution and immune cells involvement in clone selection.

Although *EGFR* and *KRAS* are closely linked within their signaling pathways, harboring oncogenic mutations in these genes differently affects prognosis, tumor biology, histological differentiation and immunogenicity (44, 45). Contrary to the immune contexture of *EGFR* and *ALK* altered tumors, the *KRAS* driven NSCLC displays predominantly IE and inflamed TME types, which suggests a different route to cancer immune evasion. The cancer-immunity cycle describes the following steps to efficient anti-tumor response: release of cancer cell antigens, antigen presentation by dendritic cells, priming and activation of T cells in lymph nodes, trafficking of effector T cells through the bloodstream, infiltration of T cells into the tumor, recognition of cancer cells by the T cells, and elimination of cancer cells (24, 27). From this perspective, *KRAS-and EGFR*-mutant tumors obviously diverge at multiple points in anti-tumor response, leading to distinct immune microenvironment phenotypes.

First, *KRAS*-mutant tumors typically exhibit a higher tumor mutational burden (TMB) generating more neoantigens which can be captured by antigen presenting cells (APC) (46). According to the study by Li T et al., *EGFR*-mutant NSCLC exhibit lower TMB (mean ~3.9 vs. ~6.1 mutations/Mb in *KRAS*, *p* < 0.001), resulting in fewer neoantigens to be available to APC to prime T cells. Next, via IL-6/JAK/STAT3 axis and NF-kB activation, *KRAS* mutant tumors promote inflammation and immune invasion. At the same time however, *KRAS* activation in lung cancer is also linked to MEK/ERK/AP-1 mediated secretion of IL-10 and TGF-β1, which regulate recruitment of CD4+, myeloid-derived suppressor cells (MDSCs) and regulatory T cells (Treg) resulting in an immunosuppressive effect via downregulation of MHC-I expression on tumor cells, preventing antigen presentation and T-cells priming (47, 48). Moreover, *KRAS*-mutant cancer cells are prone to activating cancer-associated fibroblasts (CAFs) via MAPK and PI3K signaling, promoting desmoplasia and deposition of collagen around tumor clusters (47). Such stromal rearrangements might form a barrier physically excluding T cells from penetrating into the tumor core, despite activated inflammatory IL-6 signaling and CXCR2 chemokine gradient (49). Such changes could explain the prevalence of immune excluded phenotype in *KRAS*m NSCLC. Interestingly, *EGFR* signaling can also induce extracellular matrix remodeling via PKCδ-activation which leads to enhanced collagen deposition around tumor cells, reducing T-cell entry into the tumor (50). Nevertheless, it seems that in complex with low TMB and compromised tumor antigen recognition, these changes can manifest into “immune desert” or “cold” immunophenotype, defining a poor response to immune checkpoint blockade.

Finally, co-occurring genetic alterations which typically accompany KRAS mutation in NSCLC could also impact TME. *KRAS* co-mutations with *STK11/LKB1* were shown to impair innate immune sensing by downregulating STING pathway to interferon production and diminish the expression of vascular adhesion molecule-1, thereby blocking T-cell extravasation. Additionally, the combination of *KRAS*m with *KEAP1* loss suppresses dendritic cell maturation via NRF2-mediated antioxidant programs.

Applying the concept of the cancer-immunity cycle in this study allowed us to reveal the potential mechanisms of non-responsiveness to ICI relying on low immunogenicity of tumors with altered *ALK* and *EGFR*. Most of them demonstrated ID or IE TME reflecting the inefficiency of primary tumor cell recognition.

Thus, early events in cancer cell recognition and T-cell priming are compromised. Recognition of tumor cells by the immune system relies on the functioning of APC, including dendritic cells and macrophages, able to distinguish self from nonself *via* neoantigens and the major histocompatibility complex (MHC). Further priming of T cells leads to their activation and is regulated by several antigen-processing pathways involving proteins of MHC class II and B7 (51). Expression of cytotoxic T-lymphocytes-associated antigen 4 (CTLA-4) instead of B7 contributes to immunosuppression at an early stage, defining peripheral immune tolerance and preventing T-cell activation. Indeed, activating *EGFR* mutations can upregulate signal transducer and activator of the transcription 3 (STAT3), which is involved in STAT1 regulation (52). The latter is essential for HLA1 expression. This can affect antigen presentation machinery and cancer cell recognition by APC (53). In addition, STAT3 regulates the production of various cytokines and growth factors, modulating the expression of vascular endothelial growth factor (VEGF), IL-6, and IL-10, promoting anti-inflammatory effects and inhibiting APC differentiation and maturation (54).

## Limitations of the study

This study included a limited number of cases with known genetic alterations in a confined number of genes analyzed using NGS technology. The panel utilized for NGS testing did not include such genes as *KEA*P1, *STK11*, etc. which did not allow to consider the potential impact of other genetic alterations in shaping the immunotype of tumors. No information about therapy, its efficacy and patients` outcomes was included in this study. When assessing the immune contexture of NSCLC only LUAD with alterations in *EGFR, ALK* and *KRAS* were included in the analysis. Tumors with alterations in other genes were not analyzed due to a low incidence and a small number of cases with verified oncogenic variants. Further prospective studies are required to discover molecular mechanisms defining the interplay between oncogene-driven cancer cells and immune response with respect to the tumor immunity cycle.

## Conclusion

This study revealed a high rate of *EGFR* and *ALK* rearrangements in the Ukrainian cohort of NSCLC patients with a relatively moderate frequency of *KRAS* mutations. The high rate of PD-L1 expression in NSCLC driven by *EGFR* and *ALK* alterations was accompanied by a prevalence of low immunogenicity with the prevalence of ID TME in *EGFR* mutant tumors and IE TME in *ALK-*rearranged and *KRAS* mutant tumors. Further discovery of mechanisms affecting tumor immune contexture is needed for tailoring patient management in line with particular mechanisms of immune evasion.

## Data Availability

The data presented in the study are deposited in the NCBI Sequence Read Archive (SRA), submission: SUB15436110, accession number: PRJNA1287077.

## Footnotes

^1^ https://www.medcalc.org

^2^ www.graphpad.com
