# Supplementary material for: Genetic alterations affect immune contexture of non-small cell lung cancer: Ukrainian study
Source: Front Med (Lausanne). 2025 Jul 30;12:1558016. doi: 10.3389/fmed.2025.1558016 (PMC12345293; doi:10.3389/fmed.2025.1558016)
Supplement: Supplementary file 1 [file Table_1.docx]

**Table S1. Incidence of genetic variants in oncogenes in patients with NSCLC of different sexes**

| **Genes** | **Females (n=97)** | | **Males (n=157)** | | **p** | |
| --- | --- | --- | --- | --- | --- | --- |
|  | **Absolut number** | **%** | **Absolut number** | **%** |  |  |
| ***ALK*** | 15 | 15.5% | 9 | 5.7% | | **0.014** |
| ***BRAF*** | 3 | 3.1% | 3 | 1.9% | | 0.677 |
| ***EGFR*** | 31 | 32.0% | 16 | 10.2% | | **<0.0001** |
| ***KRAS*** | 11 | 11.3% | 32 | 20.4% | | 0.084 |
| ***MET*** | 1 | 1.0% | 4 | 2.5% | | 0.652 |
| ***ROS1*** | 1 | 1.0% | 2 | 1.3% | | 1 |
| **ERBB** | 2 | 2.1% | 2 | 1.3% | | 0.637 |
| ***RET*** | 0 | 0.0% | 1 | 0.6% | | 1 |
| ***NRAS*** | 0 | 0.0% | 4 | 2.5% | | 0.3 |
| ***PIK3CA*** | 0 | 0.0% | 3 | 1.9% | | 0.289 |

* Fisher test was applied for the comparison. Bold highlights the statistically significant differences in genetic alteration rate

**Table S2. Comparison of the frequency of genetic alterations in oncogenes in NSCLC of various histological subtypes**

| **Genes** | **LUAD (n=193)** | | **SCC (n=61)** | | **p** |
| --- | --- | --- | --- | --- | --- |
|  | **Absolut number** | **%** | **Absolut number** | **%** |  |
| ***ALK*** | 23 | 11.9% | 1 | 1.6% | **0.012** |
| ***BRAF*** | 6 | 3.1% | 0 | 0.0% | 0.34 |
| ***EGFR*** | 45 | 23.3% | 2 | 3.3% | **<0.001** |
| ***KRAS*** | 34 | 17.6% | 9 | 14.8% | 0.689 |
| ***MET*** | 5 | 2.6% | 0 | 0.0% | 0.34 |
| ***ROS1*** | 3 | 1.6% | 0 | 0.0% | 1 |
| ***ERBB2*** | 3 | 1.6% | 1 | 1.6% | 0.57 |
| ***RET*** | 1 | 0.5% | 0 | 0.0% | 1 |
| ***NRAS*** | 1 | 0.5% | 3 | 4.9% | 0.044 |
| ***PIK3CA*** | 3 | 1.6% | 0 | 0.0% | 1 |
| **Total number of cases** | 193 |  | 61 |  | <0.001 |

* Fisher test was applied for the comparison. Bold highlights the statistically significant differences in genetic alteration rate.

**Table S3. PD-L1 expression in NSCLC of different histology**

| **Parameters** | **Number** | **PD-L1 negative** | **PD-L1 TPS**  **1-49%** | **PD-L1 TPS**  **>50** |
| --- | --- | --- | --- | --- |
| The whole set | 180 | 84  (46.7%0 | 62  (34.4%) | 34  (18.9%) |
| **Histological type** | | | | |
| AC | 132  (73.3%) | 57  (43.2%) | 45  (34.1%) | 30  (22.7%) |
| SCC | 48  (26.7%) | 27  (56.3%) | 17  (35.4%) | 4  (8.3%) |
| **Genetic profile** | | | | |
| Oncogene driven NSCLC | 91  (50.6%) | 31  (34.1%) | 38  (41.8%) | 22  (24.2%) |
| Non-oncogene driven NSCLC | 89  (49.4%) | 53  (59.6%) | 24  (27.0%) | 12  (13.5%) |

**Table S4. Expression of PD-L1 in *EGFR* mutated NSCLC of different histological types**

| **Genetic alterations in *EGFR*** | | | | |
| --- | --- | --- | --- | --- |
| **ALL NSCLC cases** | | | | |
| **PD-L1 status** | **Total** | ***EGFRwt*** | ***EGFRm*** | **p** |
| PD-L1 negative | 84 (46.7%0 | 78 (50.6%) | 6 (23.1%) | **0,029** |
| PD-L1  TPS 1-49% | 62 (34.4%) | 50 (32.5%) | 12 (46.2%) |  |
| PD-L1  TPS ≥50 | 34 (18.9%) | 26 (16.9%) | 8 (30.8%) |  |
| **Total** | **180** | **154 (85.6%)** | **26 (14.4%)** |  |
| **LUAD** | | | | |
| PD-L1 negative | 57 (43.2% | 53 (49.1%) | 4 (16.7%) | **0,015** |
| PD-L1  TPS 1-49% | 45 (34.1%) | 33 (30.6%) | 12 (50.0%) |  |
| PD-L1  TPS ≥50 | 30 (22.7%) | 22 (20.4%) | 8 (33.3%) |  |
| **Total** | **132** | **108 (81.8%)** | **24 (18.2%)** |  |
| **SCC** | | | | |
| Negative | 27 (56.2%) | 25 (92.6%) | 2 (7.4%) | 0.441 |
| PD-L1  TPS 1-49% | 17 (100%) | 17 (100%) | 0 |  |
| PD-L1  TPS ≥50 | 4 | 4 (100%) | 0 |  |
| **Total** | **48** | **46** | **2** |  |
| ***ALK* rearrangements** | | | | |
| **PD-L1 status** | **Total** | ***ALKwt*** | ***ALKr*** |  |
| PD-L1 negative | 84 (46.7%0 | 82 (49.1%) | 2 (15.4%) | **0.048** |
| PD-L1  TPS 1-49% | 62 (34.4%) | 54 (32.3%) | 8 (65.1%) |  |
| PD-L1  TPS ≥50 | 34 (18.9%) | 31 (18.8%) | 3 (23.1%) |  |
| **Total** | **180** | **167** | **13** |  |
| ***KRAS* alterations in NSCLC** | | | | |
| **PD-L1 status** | **Number** | ***KRASwt*** | ***KRASm*** |  |
| PD-L1 negative | 84 (46.7%0 | 68 (47.6%) | 16 (43.2%) | 0.637 |
| PD-L1  TPS 1-49% | 62 (34.4%) | 50 (35%) | 12 (32.4%) |  |
| PD-L1  TPS ≥50 | 34 (18.9%) | 25 (17.5%) | 9 (24.3%) |  |
| **Total** | **180** | **143** | **37** |  |
